# Biofilm Formation by *Mycobacterium bovis*: Influence of Surface Kind and Temperatures of Sanitizer Treatments on Biofilm Control

**DOI:** 10.1155/2014/210165

**Published:** 2014-06-01

**Authors:** Victoria O. Adetunji, Aderemi O. Kehinde, Olayemi K. Bolatito, Jinru Chen

**Affiliations:** ^1^Department of Veterinary Public Health and Preventive Medicine, University of Ibadan, Ibadan 2000005, Nigeria; ^2^Department of Medical Microbiology and Parasitology, College of Medicine, University College Hospital, University of Ibadan, Ibadan 2000005, Nigeria; ^3^Department of Food Science and Technology, The University of Georgia, Griffin, GA 30223-1797, USA

## Abstract

*Mycobacterium bovis* causes classic bovine tuberculosis, a zoonosis which is still a concern in Africa. Biofilm forming ability of two *Mycobacterium bovis* strains was assessed on coupons of cement, ceramic, or stainless steel in three different microbiological media at 37°C with agitation for 2, 3, or 4 weeks to determine the medium that promotes biofilm. Biofilm mass accumulated on coupons was treated with 2 sanitizers (sanitizer A (5.5 mg L^−1^ active iodine) and sanitizer B (170.6 g^1^ alkyl dimethylbenzyl ammonium chloride, 78 g^−1^ didecyldimethyl ammonium chloride, 107.25 g L^−1^ glutaraldehyde, 146.25 g L^−1^ isopropanol, and 20 g L^−1^ pine oil) at 28 and 45°C and in hot water at 85°C for 5 min. Residual biofilms on treated coupons were quantified using crystal violet binding assay. The two strains had a similar ability to form biofilms on the three surfaces. More biofilms were developed in media containing 5% liver extract. Biofilm mass increased as incubation time increased till the 3rd week. More biofilms were formed on cement than on ceramic and stainless steel surfaces. Treatment with hot water at 85°C reduced biofilm mass, however, sanitizing treatments at 45°C removed more biofilms than at 28°C. However, neither treatment completely eliminated the biofilms. The choice of processing surface and temperatures used for sanitizing treatments had an impact on biofilm formation and its removal from solid surfaces.

## 1. Introduction

Bovine tuberculosis (TB) caused by* Mycobacterium bovis* infection has been reported in dairy and food animals such as cattle [1–3] and goats [4]. Earlier studies have confirmed the endemicity of bovine TB in cattle herd of Nigeria with the isolation of* M. bovis* in milk [5, 6] and in lesion of farm and abattoir animals [5, 7, 8]. Although bovine TB is controlled in developed countries, a 2003–2008 retrospective study demonstrated an increasing trend in countries such as Great Britain [9].

*M. bovis* has a wide range of hosts including humans and wildlife, making it a pathogen of public health significance. Humans are usually infected with* Mycobacterium* through the inhalation of droplet nuclei; however, a significant proportion of human cases involve extrapulmonary TB, presumably caused by the consumption of raw milk from infected animals or milk and meat products contaminated with the pathogen [10]. Bovine TB caused by* M. bovis* is responsible for approximately 2,000 human deaths per annum (6%) worldwide [11] and its DNA has been identified in human remains [12]. Human-to-human transmission of the disease in HIV-positive patients has also been reported [13].

Previous studies have shown that microorganisms have a natural capacity to attach to surfaces, multiply, and embed themselves in a slimy exopolysaccharide matrix, forming biofilms. Formation of biofilm is a defense mechanism of pathogenic bacteria such as* M. bovis* against adverse environmental conditions [14–17]. The ability of bacteria to adhere to both living and nonliving surfaces and form biofilms has public health implications in a variety of fields [18–21]. Biofilm formed by* Mycobacterium* spp. is one of the concerns of food and dairy processing industry, particularly in Africa. Failure to control biofilms formed by* Mycobacterium* spp. in food processing environment could lead to recontamination of pasteurized products and cross contamination of safe foods. Ingestion of contaminated food and milk is the most significant link between bovine and human TB, particularly in children [2, 22]. This study assessed the formation of biofilms by selected strains of* M. bovis* on different contact surfaces and in various growth media. It also assessed the effectiveness of sanitizing treatments in removal of the biofilms formed by* M. bovis* from their contact surfaces.

## 2. Materials and Methods

The study was carried out in 2012 at the Tuberculosis Reference Laboratory of Department of Medical Microbiology and Parasitology, University College Hospital, Ibadan, Nigeria.

### 2.1. Bacterial Strains and Culture Conditions

Two* M. bovis* strains were used for biofilm development.* M. bovis *BCG is a reference strain (RIVM, The Netherlands) while* M. bovis* MBV1 is a bovine strain isolated in Nigeria. Both* M. bovis* strains were stored on Lowenstein-Jensen agar slants at −20°C and were subcultured, immediately prior to the experiment, in Middlebrook 7H9 broth (Remel, Lenexa, Kansas, USA) containing 10% Middlebrook growth supplement containing oleic-acid, albumin, dextrose, and catalase (OADC, Remel), 0.1% Tween 80 (Fishers Scientific, Pittsburgh, USA), and 0.2% glycerol (AnalaR, BDH Chemical Ltd., Poole, UK). Inoculated samples were incubated at 37°C aerobically with agitation for 14 d.

### 2.2. Contact Surfaces for Biofilm Development

Twelvecoupons (2 cm^2^ each) of stainless steel (West Gate, Stainless Steel, Japan), cement (PureChem Cement Ltd., Nigeria), and ceramic (Virony, Building Materials Company Ltd., China) were used as contact surfaces in the study. Prior to biofilm development, the coupons were soaked for 24 h in 5% Ariel detergent (13.5% sodium carbonate, 3.0% sodium hydroxide, 30.0% sodium sulfate, 10.0% pentasodium tripolyphosphate, 13.5% alkyl benzenesulfonic acid sodium salt, and 7.5% sodium fatty alcohol sulfate; Unilever, Lagos, Nigeria) and then rinsed 3 times with sterile distilled water each for 15 min.

The coupons were later sterilized in a hot air oven (Electro, Helios, Sweden) at 120°C for 30 min.

### 2.3. Biofilm Development

Biofilm development on various surfaces took place in 3 different microbiological media. The base medium was Middlebrook 7H9 broth with 0.1% Tween 80, which was supplemented with 5% liver extract (LE) alone, 10% OADC alone, or 5% LE plus 10% OADC. The media were prepared according to the manufacturer's instructions. The 5% LE was prepared by aseptically homogenized 5 g of fresh bovine liver tissue (Bodija Abattoir, Ibadan, Nigeria) in 10 mL of 0.9% physiologic saline (Dana Pharmaceuticals, Lagos, Nigeria). The homogenates were aseptically sieved into sterile container, and penicillin G (50 IU mL^−1^), polymyxin B (2,000 U mL^−1^), amphotericin B (200 mg mL^−1^), nalidixic acid (800 mg mL^−1^), and trimethoprim (200 mg mL^−1^) were then added.

For biofilm formation, individual stainless steel, cement, or ceramic coupon was placed in separate screw-caped glass jars (Jirui Glass Products Co. Ltd., Xuzhou, China), each containing 150 mL of one of previously described test media inoculated with either H37Rv or MTB1. Biofilm development was permitted at 37°C during a period of 2, 3, or 4 weeks with continuous agitation to increase availability of oxygen.

### 2.4. Biofilm Quantification

At the end of 2nd, 3rd, and 4th week of the incubation periods, developed biofilms were quantified using the crystal violet binding assay as previously described by Stepanović et al. [23] and Adetunji and Adegoke [24] with some modifications. At each sampling point, coupons were collected and washed 3 times, each with 5 mL of sterile distilled water. Biofilm mass was fixed with 1 mL of 95% ethanol (AnalaR, BDH Chemical Ltd., UK) for 15 min at room temperature. The fixed samples were air dried for 10 min and then stained for 5 min with 2% crystal violet (AnalaR, BDH Chemical Ltd., UK). Excess stain was rinsed with running tap water, and the coupons were then air dried. Each of the dried coupons was placed on a sterile petri dish, and 3 mL of 33% glacial acetic acid (AnalaR, BDH Chemical Ltd., UK) was used to solubilize the crystal violet. The solubilized stain was then pipetted into a cuvette (Bibby Scientific Limited, Staffordshire, UK). Absorbance (OD) readings at 600 nm were measured using a spectrophotometer (Surgienfield Instruments, Springfield, England).

### 2.5. Biofilm Control

At weeks 2, 3, and 4 sampling intervals testcoupons were collected from glass jars and washed three times with 5 mL sterile distilled water to remove loosely attached cells. The coupons were then treated for 5 min in 10 mL solution of 2% sanitizer A (5.5 mg l^−1^ active iodine; Crosley Sinbad & Co. Ltd., Nigeria) or 0.5% sanitizer B (170.6 g l^−1^ alkyl dimethylbenzyl ammonium chloride, 78 g l^−1^ didecyldimethyl ammonium chloride, 107.25 g l^−1^ glutaraldehyde, 146.25 g l^−1^ isopropanol, and 20 g l^−1^ pine oil; CID LINES, Belgium) according to the manufacturer's instructions at 28 or 45°C. Mycobacterial biofilms on tested surfaces were also treated in 10 mL water at 85°C. Thereafter, residual sanitizers were neutralized with double strength Dey-Engley solution at room temperature for 10 min followed by air dry at 60°C for 2 h, and biofilm mass on various coupons was quantified using the crystal violet binding assay described in Section 2.4.

### 2.6. Statistical Analysis

Each experiment was replicated once, and every test was duplicated. For each group of trials, 72 observations were made. Separation of means was accomplished using Fisher's least significant difference design and the General Linear Model of Statistical Analysis Software (SAS; *α* = 0.05) [25].

## 3. Results 

### 3.1. Biofilm Formation

The two* M. bovis* strains formed more biofilms on cement than on ceramic and stainless steel surface after a 2-week incubation period at 37°C (Table 1). Strain MBV1 formed more biofilm on ceramic than on stainless steel surface in broth containing 5% LE and 5% LE plus 10% OADC; however, similar phenomena were not observed with strain BCG (Table 1). Furthermore, strain MBV1 formed more biofilms than BCG on the three surfaces in all media except on one occasion in broth containing 10% OADC plus 5% LE on stainless steel surface (Table 1).

When the incubation time was extended to 3 weeks, an increase in biofilm mass was observed (Tables 1 and 2). Similar to the trend observed in week 2, both strains of* M. bovis* formed more biofilms on cement than on ceramic and stainless steel surfaces (Table 2). Strain BCG formed more biofilms on stainless steel than ceramic surface in all growth media. However, MBV1 formed more biofilms on ceramic than stainless surface in broth containing 5% LE and 5% LE plus 10% OADC (Table 2).

At the 4th week sampling point it became clear that significantly more biofilms were formed by the two* M. bovis* strains on cement than on ceramic and stainless steel surface (Table 3). Different from what was observed in week 3, BCG formed more biofilms on ceramic than on stainless steel surface in all growth media. Similar to what was observed in week 3, MBV1 formed more biofilms on ceramic than on stainless steel surface in broth containing 5% LE plus 10% OADC. In broth containing 5% LE, however, MBV1 formed significantly less biofilms on ceramic than on stainless steel surface (Table 3).

Results of overall statistical analysis revealed that the amount of biofilm mass increased as the incubation time increased from week 2 to week 3. However, the biofilm mass remained essentially the same when the incubation time was extended to the 4th week (Table 4). The two* M. bovis* strains formed more biofilms on cement than on ceramic and stainless steel surfaces (Table 4). Furthermore, broth with LE and OADC better supported biofilm formation than broth with a single supplement. There is no statistical difference in biofilm mass formed in the two microbiological media that contained a single supplement (Table 4). Overall, strain MBV1 and BCG had a similar ability in forming biofilms (Table 4).

### 3.2. Effect of Sanitizing Treatments on* M. bovis* Biofilms

Treatment with hot water significantly reduced the amount of biofilms on coupons. However, greater reductions in biofilm mass were observed when coupons were treated by 2% sanitizer A and 0.5% sanitizer B (Table 5). Treatments performed at 45°C removed more biofilms than the treatments at 28°C (Table 5). Treatments with 0.5% sanitizer B were more effective than those with 2% sanitizer A at 28°C. However, opposite results were observed at 45°C (Table 5).

## 4. Discussion

### 4.1. Biofilm Forming Potential of* M. bovis* Strains

A previous study has shown that* M. tuberculosis* could develop biofilms on polystyrene surface [26]. However, mycobacterial biofilms developed on cement, stainless steel, and ceramic surfaces have not been studied previously. The present study represents our attempt to assess biofilm development by* M. bovis* on these surfaces. Results of the research indicated that both strains of* M. bovis *used in the study were capable of forming biofilms. These findings were consistent with those of some of the earlier observations [17, 26, 27]. The present study found that a greater amount of biofilm developed on cement surface than on ceramic and stainless steel surfaces (Tables 1–3). Earlier studies have suggested that the topography of bacterial contact surface plays an important role in facilitating bacteria adhesion and biofilm formation [28–32]. The roughness of a bacterial contact surface has also been suggested to enhance the attachment of bacterial cells [33–35]. A previous study found that significantly higher bacterial densities were associated with biofilms formed on cement-lined pipes than on galvanized steel pipes [36].

The current study demonstrated that extending incubation time from 2 to 3 weeks allowed more biofilm mass to accumulate (Table 4). The reason for the decrease in biofilm mass from week 3 to week 4 is currently unknown. However, nutrient depletion and accumulation of bacterial waste and metabolic products in the growth media might be responsible for the observed phenomenon. Nutrient that induced biofilm dispersion has been observed in* Pseudomonas aeruginosa* PAO1 in earlier studies [37].

One of the important goals of the present study was to assess the role of iron in* M. bovis* biofilm development. Results of the study demonstrated that cells of* M. bovis* formed more biofilms in broth containing the iron-rich LE (Table 4). The enhancement of mycobacterial biofilm formation by elevated iron concentration has been noticed by several previous studies [26, 38, 39]. It is believed that iron is an important cofactor which plays a central role in biofilm development [38–40]. Supplemental iron above 1 *μ*M has been shown to be necessary for biofilm development by* M. smegmatis* although iron is not needed for planktonic growth [38].

### 4.2. Control of* M. bovis* Biofilms

We evaluated the effectiveness of a few selected treatments in control of* M. bovis* biofilms. Results of the study demonstrated that treatments with the two chemical sanitizers significantly reduced the amount of biofilm mass on mycobacterial contact surfaces used in the present study (Table 5). Sanitizer B is significantly (*P* < 0.05) more effective than sanitizer A at 28°C. However, opposite phenomenon was observed at 45°C. Sanitizer B is a polyvalent sanitizer composed of two quaternary ammonium compounds, alkyl dimethylbenzyl ammonium chloride, and didecyldimethyl ammonium chloride, as well as aldehyde and pine oil. The bactericidal action of the quaternaries has been linked to the inactivation of energy-producing enzymes, denaturation of essential cell proteins, and disruption of cell membranes [41]. Sanitizer A is a monovalent sanitizer composed of active iodine [42]. The bactericidal activity of iodine is accomplished by direct halogenation of proteins, damage of cell walls, and destruction of enzyme activities [42]. Consistent with findings of this study, previous reports have found that the effectiveness of iodine increased at higher temperatures [43, 44].

Sanitizing treatments at 45°C were significantly (*P* < 0.05) more effective than those at 28°C (Table 5). It seems plausible that, at higher temperatures, the sanitizers potentiated their disinfection and dislodging effects on the biofilms, thus producing a drastic reduction in biofilm mass on their contact surfaces. Similar to the present study, an earlier report demonstrated that biofilms were completely removed from the surfaces of a model copper water plumbing system operated at 60°C [45]. In a separate study, a mycobacterial biofilm mass on plastic surfaces was not affected by treatment with water at 22 to 30°C [46].

Neither treatment used in the present study eliminated the biofilms from mycobacterial contact surfaces (Table 5). Earlier studies have shown that biofilm embedded cells are more persistent than planktonic cells [47]. The biofilm matrices could reduce the rate of sanitizer diffusion, and, as a result, only the biofilm mass close to the surface were reached by the sanitizers while the deeper layers of the biofilms remained unaffected.

## 5. Conclusion

Both strains of* M. bovis* used in the present study developed biofilms on cement, ceramic, and stainless steel surfaces. In general, cells formed more biofilms on cement than on ceramic and stainless steel surfaces. Growth media containing the iron-rich LE significantly enhanced biofilm formation. The study suggests that careful selection of food processing materials and good environmental hygiene are important considerations in terms of biofilm control. It also suggests that raising the temperature of sanitizing treatments will generate a greater biofilm reduction.

## Figures and Tables

**Table 1:** Biofilms (OD 600_nm_) formed by *Mycobacterium bovis* strains after 2 weeks of incubation in three different microbiological media at 37°C.

Base growth media (7H9 with 0.1% T80) and supplements	*Mycobacterium bovis * strains	Biofilm mass (*A*_600_nm__) (*n* = 4)		
		Stainless steel	Cement	Ceramic
5% liver extract	BCG	0.603^bF^	0.623^aF^	0.614^abE^
	MBV1	0.667^cD^	1.119^aB^	0.862^Bb^
10% OADC and 5% liver extract	BCG	0.984^bA^	1.105^aC^	0.852^cC^
	MBV1	0.961^cB^	1.128^aA^	0.972^bA^
10% OADC	BCG	0.616^bE^	0.648^aE^	0.617^bE^
	MBV1	0.807^aC^	0.809^aD^	0.733^bD^

OADC: a growth supplement containing oleic, albumin, dextrose, and catalase.

Means in the same row not followed by the same lowercase letter are statistically significant (*P* < 0.05).

Means in the same column not followed by the same uppercase letter are statistically significant (*P* < 0.05).

**Table 2:** Biofilms (OD 600_nm_) formed by *Mycobacterium bovis* strains after 3 weeks of incubation in three different microbiological media at 37°C.

Base growth media (7H9 with 0.1% T80) and supplements	*Mycobacterium bovis * strains	Biofilm mass (*A*_600_nm__) (*n* = 4)		
		Stainless steel	Cement	Ceramic
5% liver extract	BCG	1.118^bD^	1.145^aC^	1.112^cD^
	MBV1	1.238^cB^	1.477^aAB^	1.339^bB^
10% OADC and 5% liver extract	BCG	1.150^bC^	1.481^aAB^	0.593^cE^
	MBV1	1.019^cF^	1.500^aA^	1.439^bA^
10% OADC	BCG	1.048^bE^	1.464^aAB^	0.409^cF^
	MBV1	1.338^bA^	1.475^aAB^	1.317^bC^

OADC: a growth supplement containing oleic, albumin, dextrose, and catalase.

Means in the same row not followed by the same lowercase letter are statistically significant (*P* < 0.05).

Means in the same column not followed by the same uppercase letter are statistically significant (*P* < 0.05).

**Table 3:** Biofilms (OD 600_nm_) formed by *Mycobacterium bovis* strains after 4 weeks of incubation in three different microbiological media at 37°C.

Base growth media (7H9 with 0.1% T80) and supplements	*Mycobacterium bovis * strains	Biofilm mass (*A*_600_nm__) (*n* = 4)		
		Stainless steel	Cement	Ceramic
5% liver extract	BCG	0.887^cA^	1.733^aA^	1.679^bA^
	MBV1	0.723^bB^	1.475^aAB^	0.557^cF^
10% OADC and 5% liver extract	BCG	0.723^cB^	1.793^aA^	1.456^bB^
	MBV1	0.685^cC^	1.477^aAB^	0.958^bD^
10% OADC	BCG	0.686^cC^	1.785^aA^	1.276^bC^
	MBV1	0.332^bD^	1.472^aC^	0.714^abE^

OADC: a growth supplement containing oleic, albumin, dextrose, and catalase.

Means in the same row not followed by the same lowercase letter are statistically significant (*P* < 0.05).

Means in the same column not followed by the same uppercase letter are statistically significant (*P* < 0.05).

**Table 4:** Influence of various factors on the formation of biofilms by strains of *Mycobacterium bovis*.

	Biofilm mass (*A*_600_)
Incubation time (*n* = 72)	
Week 2	0.818^B^
Week 3	1.200^A^
Week 4	1.134^A^
Cell contact surface (*n* = 72)	
Cement	1.317^A^
Ceramic	0.927^B^
Stainless steel	0.866^C^
Growth supplement (*n* = 72)	
Liver extract and OADC	1.126^A^
Liver extract alone	1.054^AB^
OADC alone	0.975^B^
Bacterial strain (*n* = 108)	
BCG	1.059^A^
MBV1	1.044^A^

OADC: a growth supplement containing oleic-acid, albumin, dextrose, and catalase. Means in the same column not followed by the same upper case letters is significantly different (*P* < 0.05).

**Table 5:** Efficacy of sanitizing treatments on biofilm removal.

Treatment	Temperature (°C)	Residual biofilm mass (*A*_600_)
Control (*n* = 216)		1.027^a^
Water (*n* = 216)	85	0.762^b^
Sanitizer A (2%; *n* = 432)	28	0.661^c^
	45	0.428^f^
Sanitizer B (0.5%; *n* = 432)	28	0.633^d^
	45	0.432^e^

Means in the same column not followed by the same lower case letters is significantly different (*P* < 0.05).
